# Synergistic Effect of Combinations Containing EDTA and the Antimicrobial Peptide AA230, an Arenicin-3 Derivative, on Gram-Negative Bacteria

**DOI:** 10.3390/biom8040122

**Published:** 2018-10-23

**Authors:** Anita Umerska, Magnus Strandh, Viviane Cassisa, Nada Matougui, Matthieu Eveillard, Patrick Saulnier

**Affiliations:** 1 Université de Lorraine, CITHEFOR, F-54000 Nancy, France; 2 MINT, UNIV Angers, INSERM 1066, CNRS 6021, Université Bretagne Loire, F-49933 Angers, France; matougui.nada@gmail.com (N.M.); patrick.saulnier@univ-angers.fr (P.S.); 3 Adenium Biotech ApS, DK-2200 Copenhagen, Denmark; mst@adeniumbiotech.com; 4 Laboratoire de bactériologie, 49100 CHU Angers, France; vivianecassisa@yahoo.fr; 5 Equipe ATIP AVENIR, CRCINA, Inserm, Université de Nantes, Université d’Angers, 49100 Angers, France; MaEveillard@chu-angers.fr

**Keywords:** antimicrobial peptides, synergy, arenicin, EDTA, gram-negative bacteria, antibiotic resistance, *Acinetobacter baumannii*, *Escherichia coli*, *Pseudomonas aeruginosa*, ESKAPE pathogens

## Abstract

The worldwide occurrence of resistance to standard antibiotics and lack of new antibacterial drugs demand new strategies to treat complicated infections. Hence, the aim of this study was to examine the antibacterial activities of an antimicrobial peptide, arenicin-3 derivative AA230, and ethylenediaminetetraacetic acid (EDTA) as well as the two compounds in combination against Gram-negative bacteria. AA230 showed strong antibacterial activity against all of the studied standard strains and clinical isolates, with minimum inhibitory concentrations ranging between 1 µg/mL and 8 µg/mL. AA230 exhibited a bactericidal mode of action. EDTA inhibited the growth of *Acinetobacter baumannii* at 500–1000 µg/mL. Strains of *Acinetobacter baumannii* were found to be more susceptible to EDTA than *Pseudomonas aeruginosa* or *Escherichia coli*. The antibacterial effects of both AA230 and EDTA were independent of the antibiotic resistance patterns. Indifference to synergistic activity was observed for AA230 and EDTA combinations using checkerboard titration. In time-kill studies, a substantial synergistic interaction between AA230 and EDTA was detected against all of the tested strains. The addition of EDTA enabled a 2–4-fold decrease in the AA230 dose. In conclusion, AA230 could have potential applications in the treatment of infections caused by Gram-negative organisms, and its effect can be potentiated by EDTA.

## 1. Introduction

The widespread use of antibiotics has led to the emergence of many resistant pathogenic microorganisms [1]. Resistance to antibiotics continues to be a major problem with an increasing number of hospitalizations and deaths attributed to resistant organisms each year. It is essential to combat this threat by developing new antibiotics and new formulations [2,3,4].

Recently, antimicrobial peptides have attracted the attention of scientists and pharmaceutical companies as possible alternatives to conventional antibiotics [5,6,7,8]. These peptides are considered to play a key role in the host defense systems of most multicellular organisms, including humans [1,9]. Antimicrobial peptides generally contain between 12 and 50 amino acids with a large proportion (generally above 50%) of hydrophobic amino acids and two or more positively charged residues, including arginine, lysine or, in acidic environments, histidine [10,11,12]. Antimicrobial peptides exert bactericidal activity by increasing the permeability of the cytoplasmic membranes of targeted organisms, although they may have additional intracellular targets [1,3,12,13,14]. In addition to killing bacteria directly, they have a number of immunomodulatory functions [15].

Arenicin-3 is a novel antimicrobial peptide that was isolated from the lugworm *Arenicola marina*. It is composed of 21 amino acids (GFCWYVCVYRNGVRVCYRRCN) with two disulphide bonds bridging Cys3 and Cys20 and Cys7 and Cys16. Arenicin-3 and its analogues exhibit potent and rapid antimicrobial activity in vitro against a broad range of multi-resistant pathogenic Gram-negative bacteria, including polymyxin resistant *Pseudomonas aeruginosa*, *Acinetobacter baumannii* and *Klebsiella pneumoniae*. The peptide also demonstrated in vivo activity in urinary tract infection (UTI) and pneumonia mouse models [16]. Cytotoxicity and haemolysis assays were performed for arenicin-3 derivatives using HEK293 cells and human red blood cells, respectively [16]. The peptides did not display haemolytic activity at 300 µg/mL, and the IC_50_ towards HEK293 cells was ≥100 µM for the majority of tested peptides. The study by Wang et al. [17] showed that an arenicin-3 derivative, NZ17074, exerted antifungal properties in addition to its antibacterial activity.

Combined administration of antibacterial agents has gained interest because it often leads to synergistic effects. Administering synergistic combinations could be a way to overcome problems with toxicity and the development of resistance. Several studies on the synergistic effects of antimicrobial peptides, including arenicin-1, with conventional antibiotics have been reported [18]. In addition to conventional antibiotics, potentiating and sensitizing agents such as ethylenediaminetetraacetic acid (EDTA) can prove useful in increasing the efficacy of antimicrobial agents.

EDTA is a chelating agent that exhibits high affinity for metal ions and a high density of ligands with binding typically occurring through its two amino and four carboxylate groups [19]. EDTA has the ability to “sequester” metal ions such as Ca^2+^ and Fe^3+^. After being bound by EDTA in a metal complex, metal ions remain in solution but exhibit diminished reactivity. In the manufacturing of pharmaceutical products, EDTA is sometimes added to improve the stability as EDTA can prevent metal-catalyzed redox or free radical reactions by binding metals. EDTA is used extensively for the treatment of patients who have been poisoned with heavy metal ions such as lead and mercury [19]. EDTA is not recognized as an important antimicrobial agent in its own right. It is generally regarded as a ‘potentiator’ of the activity of other antimicrobial agents [20,21]. EDTA has been shown to exhibit synergistic interactions with a 12-residue cationic antimicrobial peptide ARVA [3].

The aim of this in vitro study was to examine the antibacterial activity of the arenicin-3 derivative AA230 and EDTA by determining minimum inhibitory concentrations (MIC) and minimum bactericidal concentrations (MBC) with further investigations into the modes of action of these molecules using time kill assays. Another important objective was to investigate the impact of EDTA on the antibacterial activity of AA230 against Gram-negative bacteria, including *Pseudomonas aeruginosa*, *Escherichia coli* and *Acinetobacter baumannii*, as well as antibiotic resistant strains of these bacteria.

## 2. Materials and Methods

### 2.1. Materials

Antimicrobial peptide AA230 (molecular weight 2578 kDa, theoretical isoelectric point 9.69) was synthesized and provided by PolyPeptide Laboratories (Limhamn, Sweden) in the form of a lyophilized chloride salt (chloride content 6.8% as determined by ion exchange chromatography). Ethylenediaminetetraacetic acid (EDTA) dihydrate disodium salt was purchased from Sigma Aldrich (Saint-Quentin Fallavier, France). Brain–heart infusion (BHI) broth was purchased from bioMérieux (Marcy l’Étoile, France). Columbia agar supplemented with sheep blood (5%) was obtained from Oxoid (Dardilly, France). All other chemicals and solvents were of analytical grade.

### 2.2. Bacterial Strains

Antibacterial activity was tested against three reference strains, *Pseudomonas aeruginosa* (ATCC 27853), *Escherichia coli* (ATCC 25922) and *Acinetobacter baumannii* AYE (ATCC BAA-1710), as well as three clinical isolates, *Pseudomonas aeruginosa* (0704C0134 resistant to piperacillin/tazobactam, ticarcillin, ciprofloxacin and levofloxacin), an extended-spectrum beta-lactamase (ESBL) *Escherichia coli* (9007550201) and *Acinetobacter baumannii* RCH, that were obtained from the University Hospital of Angers (France).

### 2.3. Preparation of Inoculum

The test strains were grown overnight on Columbia agar at 37 °C. Bacterial suspensions with a density of 0.5 McFarland were prepared in 0.85% NaCl and diluted 10-fold with BHI.

### 2.4. MIC and MBC

MIC and MBC were determined via a broth microdilution method [22,23]. The compound formulations were diluted with BHI in poly-propylene tubes. One hundred microliters of BHI was added to each well of a sterile 96-well plate. Serial two-fold dilutions of the samples were performed in BHI to obtain the desired concentration range. One hundred microliters of a bacterial suspension in BHI was added to each well. The plates were incubated at 37 °C for 24 h without shaking. The MIC was defined as the lowest concentration of the tested compound that inhibited visible bacterial growth. To determine the MBC, approximately 3 μL was withdrawn from each well using a stainless steel 96-well plate replica plater (8 × 12 array), transferred onto a plate containing Mueller Hinton agar and incubated overnight at 37 °C. The lowest concentration that did not show bacterial growth was considered to be the MBC. MIC and/or MBC values were considered different if they varied by more than one dilution [22].

### 2.5. Checkerboard Titration

Combinations of AA230 and EDTA were tested via a checkerboard titration method. Two-fold dilutions of AA230 and EDTA were prepared before mixing. The concentration EDTA ranged from 8 to 512 µg/mL, whereas the concentration of AA230 varied between 0.0625 µg/mL and 64 µg/mL. The fractional inhibitory concentration (FIC) index was calculated using the following equation:

(1) $$FIC=\frac{MIC_{AA230\;in\;combination}}{MIC_{AA230\;alone}}+\frac{MIC_{EDTA\;in\;combination}}{MIC_{EDTA\;alone}}$$

Synergy was defined as an FIC index of ≤0.5 and additivity/indifference as an FIC index of >0.5 but of ≤4. Antagonism was defined as an FIC index of >4 [24].

### 2.6. Time-Kill Studies

The time-kill studies were performed with a final inoculum of approximately 1.5 × 10^5^ CFU/mL in a final volume of 2 mL in a polypropylene tube. The samples and the control (a bacterial suspension in BHI) were incubated at 37 °C. At each sampling time (0, 3, 6, 18 and 24 h), 100 μL was withdrawn from each tube, and serial 100-fold dilutions were prepared in distilled water when necessary. A 100-μL aliquot of the diluted and/or undiluted sample was delivered onto the surface of the agar. After incubating the agar plates for 24 h at 37 °C, the colonies were counted.

The activity was considered to be bactericidal when the original inoculum was reduced by ≥3 log CFU/mL (99.9%), and bacteriostatic activity was defined as a reduction in the original inoculum by <3 log CFU/mL [25,26]. Synergy was defined as a >2 log decrease in the number of CFU per mL compared with the most active single agent [26,27].

## 3. Results

### 3.1. Antibacterial Activity of AA230

Table 1 shows the antibacterial activity of the arenicin-3 derivative AA230. This peptide showed potent activity against all tested strains, including the antibiotic resistant phenotypes, with MICs ranging from 1 µg/mL to 8 µg/mL, depending on the strain. For each strain, the difference between the lowest MIC and the highest MIC or MBC was 2-dilutions (4-fold); therefore, AA230 is bactericidal. Interestingly, at higher concentrations (64–128 µg/mL), AA230 also inhibited the growth of two strains of Gram-positive *Staphylococcus aureus*, including a methicillin-resistant strain (data not shown).

**Figure 1:**
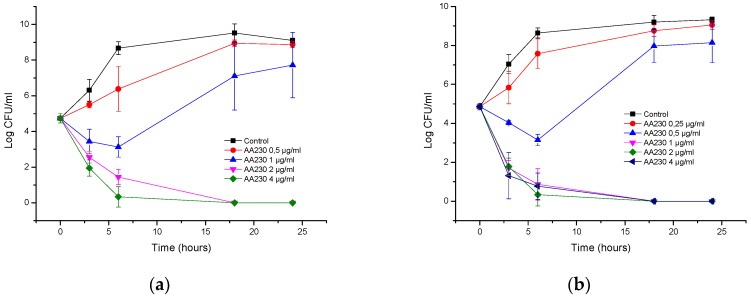
Time-kill curves of antimicrobial peptide AA230 against (**a**) *Escherichia coli* ATCC (American Type Culture Collection); (**b**) *Escherichia coli* ESBL (extended spectrum beta lactamase).

AA230 at 2 µg/mL (MIC) and 4 µg/mL (2 × MIC) demonstrated bactericidal activity after 18–24 h against the reference strain of *E. coli* (Figure 1a). At 1 µg/mL (0.5 × MIC), initial growth inhibition was followed by bacterial regrowth after 18 and 24 h. Time-kill assays showed that the ESBL *E. coli* strain was more susceptible to AA230 than the ATCC 25922 strain (Figure 1b). A rapid bactericidal effect (a CFU reduction equal to or higher than 3 log CFU/mL) was observed at the following concentrations for the ESBL strain: 1 µg/mL (MIC), 2 µg/mL (2 × MIC) and 4 µg/mL (4 × MIC). At 0.5 µg/mL (0.5 MIC), initial inhibition of bacterial growth was observed after 18 and 24 h.

**Figure 2:**
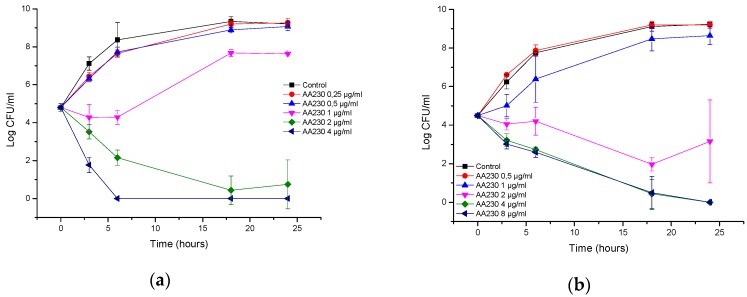
Time-kill curves of antimicrobial peptide AA230 against (**a**) *Acinetobacter baumannii* ATCC (American Type Culture Collection); (**b**) *Acinetobacter baumannii* RCH.

In the case of *A. baumannii* ATCC strain BAA-1710, bactericidal action was observed after 3 h at 4 µg/mL (2 × MIC), whereas at 2 µg/mL (MIC), a bactericidal effect was observed after 18 h (Figure 2a). Bacterial killing occurred more slowly with the RCH strain; at 8 µg/mL (4 × MIC) and 4 µg/mL (2 × MIC), the reduction in CFUs was approximately 1.9 log CFU/mL after 6 h, and a bactericidal effect was observed after 18 h (Figure 2b). At 2 µg/mL (MIC), a bacteriostatic effect was observed after 18 h with the lowest reduction in CFUs, which was 2.5 log CFU/mL compared with the starting inoculum.

**Figure 3:**
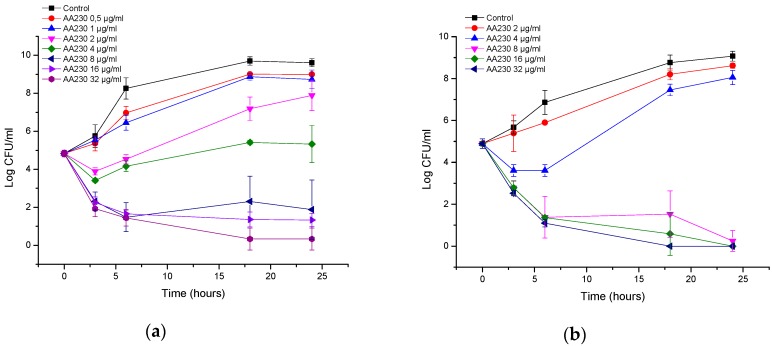
Time-kill curves of antimicrobial peptide AA230 against (**a**) *Pseudomonas aeruginosa* ATCC (American Type Culture Collection); (**b**) *Pseudomonas aeruginosa* clinical isolate.

At 8–32 µg/mL (2–8 × MIC), AA230 proved to be bactericidal against the reference strain of *P. aeruginosa* after 3–6 h of incubation (Figure 3a). At 4 µg/mL (MIC), AA230 exerted a bacteriostatic effect. In the clinical strain of *P. aeruginosa*, a bactericidal effect was observed after 3–6 h at concentrations of 8–32 µg/mL (2–8 × MIC) (Figure 3b). At 4 µg/mL (MIC), an initial reduction in the CFUs was followed by bacterial growth; however, the number of CFUs after 24 h was reduced by 1 log compared to the control.

### 3.2. Antibacterial Activity of EDTA

When used alone, EDTA was not bactericidal at any tested concentration (MBC >16 mg/mL for all tested strains). EDTA was found to inhibit the growth of *A. baumannii*; the reference and clinical strains were inhibited at 512 µg/mL and 1024 µg/mL, respectively. Only very high concentrations of EDTA were capable of inhibiting the growth of *P. aeruginosa* (MIC = 8 mg/mL), but the growth of *E. coli* was inhibited at an even higher concentration (MIC ≥ 16 mg/mL). Therefore, bacteria can be ranked in the following order based on their decreasing sensitivity to EDTA: *A. baumannii* > *P. aeruginosa* > *E. coli*.

**Figure 4:**
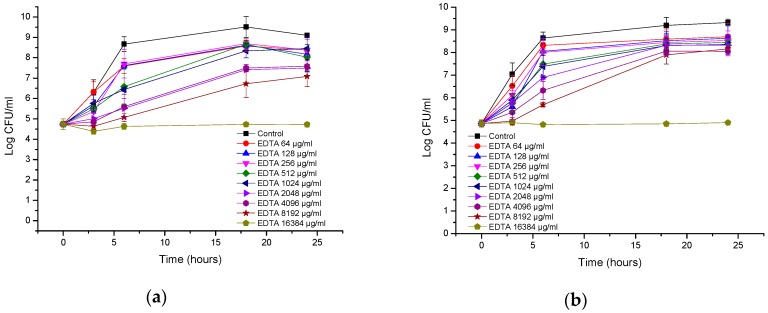
Time-kill curves of EDTA (etlylenediaminetetraacetic acid) against (**a**) *Escherichia coli* ATCC (American Type Culture Collection); (**b**) *Escherichia coli* ESBL (extended spectrum beta lactamase).

Figure 4a,b show that both *E. coli* strains were resistant to EDTA, and only at the highest tested concentration (16 mg/mL) was the growth of *E. coli* inhibited, showing a bacteriostatic effect. At lower concentrations, growth was observed, but depending on the concentration, the growth was slightly less than that of control.

**Figure 5:**
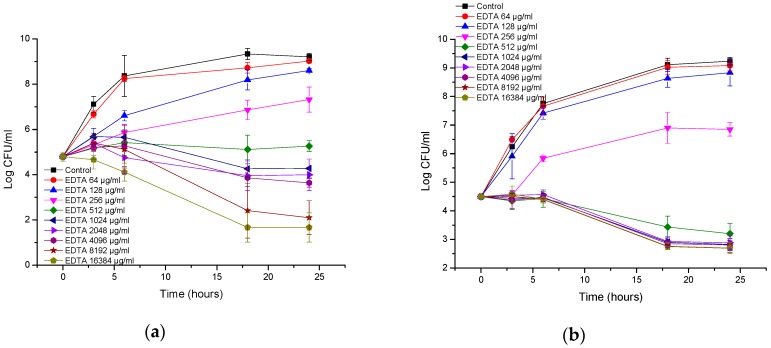
Time-kill curves of EDTA against (**a**) *Acinetobacter baumannii* ATCC (American Type Culture Collection); (**b**) *Acinetobacter baumannii* RCH.

EDTA inhibited the growth of the reference strain of *A. baumannii* (Figure 5a). The effect can generally be considered to be bacteriostatic. Only at the highest concentration of 16 mg/mL was a 3 log CFU/mL reduction observed, indicating bactericidal action at this concentration. Interestingly, the MIC/MBC assays did not show bactericidal action at 16 mg/mL of EDTA, which can be attributed to different inoculum sizes. The extent of growth inhibition was dependent on the concentration of EDTA. At high concentrations, EDTA also inhibited the growth of a clinical *A. baumannii* isolate (Figure 5b). The effect was bacteriostatic, and the maximal reduction in the number of CFUs was 1.8 log CFU/mL after 18–24 h. Interestingly, the 1–16 mg/mL concentrations showed the same inhibition profiles. Hence, there is no benefit in increasing the EDTA concentration above 1 µg/mL.

**Figure 6:**
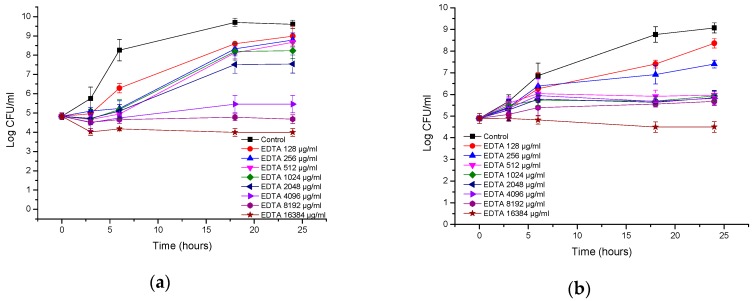
Time-kill curves of EDTA against (**a**) *Pseudomonas aeruginosa* ATCC; (**b**) *Pseudomonas aeruginosa* clinical isolate.

The growth of *P. aeruginosa* was also inhibited at high EDTA concentrations, and the inhibition was dose-dependent (Figure 6a). EDTA showed a bacteriostatic effect against the ATCC strain at 8 and 16 mg/mL; at 4 mg/mL, an increase in CFUs of 0.7 log CFU/mL compared with the initial inoculum was observed after 24 h. EDTA showed a bacteriostatic effect against the clinical isolate of *P. aeruginosa* at 16 mg/mL; at concentrations of 0.5-8 mg/mL, a small increase in CFUs of 0.8–1.0 log CFU/mL compared with the initial inoculum was observed after 18–24 h (Figure 6b).

### 3.3. Antibacterial Activity of AA230/EDTA Combinations

To determine whether AA230 can interact with EDTA to have an enhanced antibacterial effect, a checkerboard analysis was carried out. The MICs of both ingredients used in combination are shown in Table 2. Synergistic or additive interactions were observed between AA230 and EDTA against all tested strains, with FIC index values between 0.129–1.016. The interactions were examined in more detail using time-kill assays.

**Figure 7:**
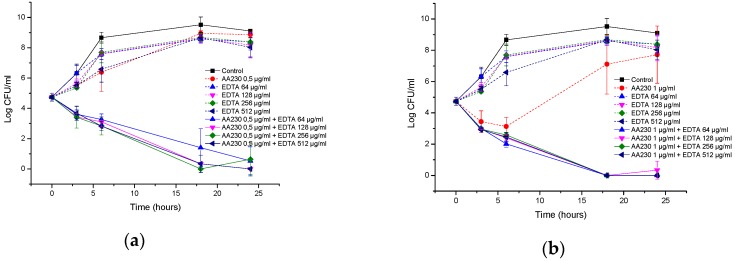

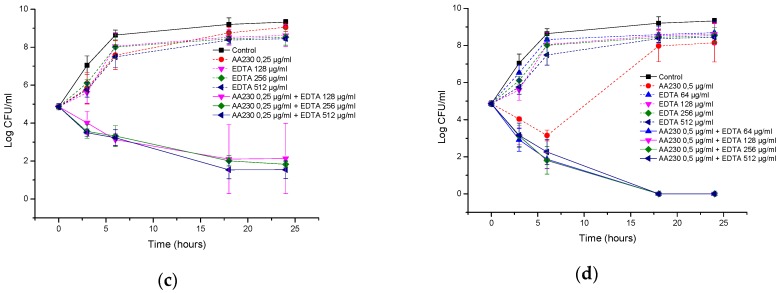
Time-kill curves of antimicrobial peptide AA230 and EDTA used alone and in combination against (**a**,**b**) *Escherichia coli* ATCC; (**c**,**d**) *Escherichia coli* ESBL.

Figure 7a,b show the time-kill curves of AA230 and EDTA and their combinations against the reference strain of *E. coli*. When the compounds were used alone, bacterial growth was observed. Combinations containing AA230 (either at 1 µg/mL or at 0.5 µg/mL) and 64–512 µg/mL of EDTA showed synergistic effects and had similar time-kill profiles, with a bactericidal effect observed after 18–24 h. Similar to the reference strain of *E. coli*, growth of the ESBL *E. coli* was observed when the compounds were used alone (Figure 7c,d). Synergy was observed when AA230 was used in combination with EDTA. The shape of the inhibition profiles depended on the content of AA230-samples containing 0.5 µg/mL of AA230 and EDTA yielded a reduction in CFUs of 4.9 log CFU/mL after 18–24 h, whereas samples containing 0.25 µg/mL of AA230 reduced CFUs by 2.7–3.3 log CFU/mL after 18–24 h. The use of EDTA enabled a 4-fold reduction in the AA230 dose in both tested *E. coli* strains.

**Figure 8:**
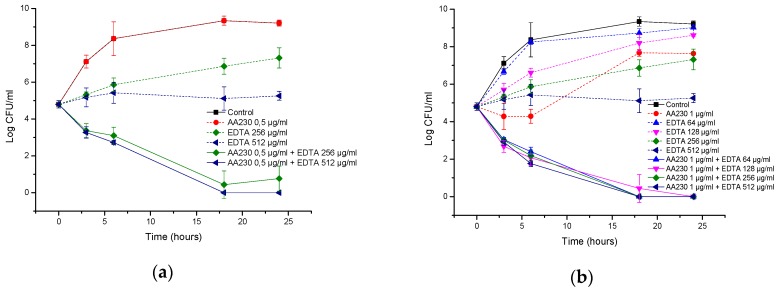

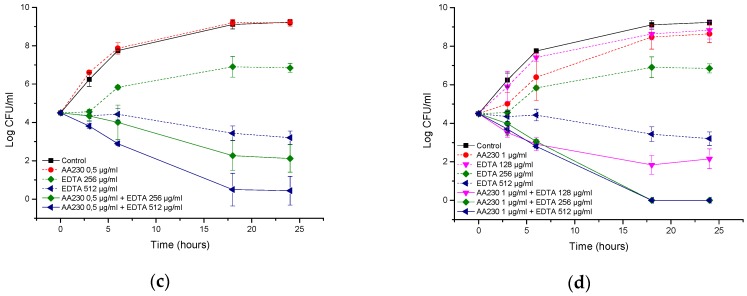
Time-kill curves of antimicrobial peptide AA230 and EDTA used alone and in combination against (**a**,**b**) *Acinetobacter baumannii* ATCC; (**c**,**d**) *Acinetobacter baumannii* RCH.

In the *A. baumannii* reference strain, EDTA alone at 512 µg/mL showed a bacteriostatic effect, but its combination with AA230 (0.5 µg/mL or 1 µg/mL) showed synergistic interactions and produced bactericidal effects after 18–24 h (Figure 8a,b). When the EDTA concentration was decreased to 256 µg/mL, synergistic effects were still observed for AA230 at both 0.5 µg/mL and at 1 µg/mL. When the EDTA concentration was further decreased to 128 µg/mL or 64 µg/mL, a synergistic and bactericidal effect was observed only in samples containing AA230 at 1 µg/mL; in combinations with a lower AA230 concentration (0.5 µg/mL), bacterial growth was observed (not shown).

Similar to the reference strain, in a clinical strain of *A. baumannii*, EDTA alone at 512 µg/mL produced a bacteriostatic effect, whereas its combination with AA230 (0.5 µg/mL or 1 µg/mL) showed synergistic and bactericidal effects (Figure 8c,d). The combination containing AA230 at 1 µg/mL and EDTA at 256 µg/mL also produced a synergistic and bactericidal effect. Sample containing 128 µg/mL EDTA and 1 µg/mL produced a bacteriostatic and synergistic effect (the difference between the compound combination and the compounds alone was more than 6 log CFU/mL). The combination containing AA230 at 0.5 µg/mL and EDTA at 256 µg/mL produced a bacteriostatic and synergistic effect (more than a 4 log CFU/mL difference between the combination of the compounds and the most active ingredient, EDTA, at 256 µg/mL). Interestingly, this combination proved to be less effective at killing than those containing higher quantities of either AA230 or EDTA.

**Figure 9:**
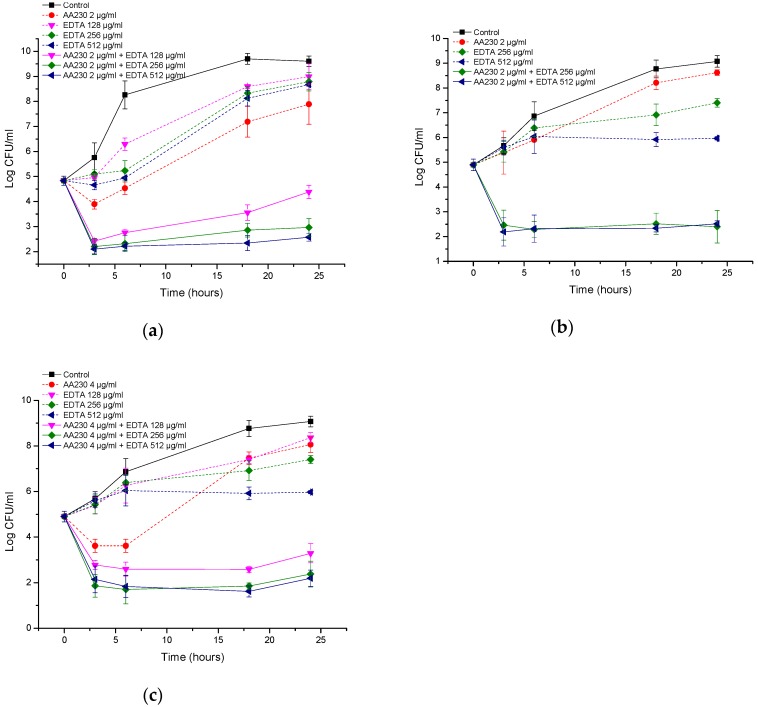
Time-kill curves of antimicrobial peptide AA230 and EDTA used alone and in combination against (**a**) *Pseudomonas aeruginosa* ATCC; (**b**,**c**) *Pseudomonas aeruginosa* clinical isolate.

In the case of the reference strain of *P. aeruginosa*, a reduction in CFUs of 2.4–2.7 log CFU/mL was observed after three hours of incubation for the AA230/EDTA combinations (Figure 9a). Similarly, in the clinical isolate of *P. aeruginosa*, a reduction in CFUs of 2.1–3 log CFU/mL was observed within three hours of incubation with the combinations (Figure 9b,c). All tested combinations can be considered synergistic (more than 2 log CFU/mL differences were observed between the combination and the most active ingredient alone after 24 h of incubation). The behaviors of the AA230 and EDTA combinations against *P. aeruginosa* were slightly different than against *E. coli* or *A. baumannii* in that after approximately three hours of incubation, a maximal reduction in CFUs occurred, and the effect was either maintained or a slight increase in CFUs was observed later.

## 4. Discussion

One of the aims of this study was to examine the antibacterial properties of an arenicin-3 derivative, AA230. AA230 showed strong antibacterial activity against all studied reference strains and clinical isolates. Because arenicin-3 is an amphipathic molecule rich in arginine and hydrophobic amino acids, killing of bacteria involves the disruption of the membrane [28]. Indeed, the MBC and time-kill assay results confirmed the bactericidal action of AA230. A detailed study on the mechanism of action of arenicin-3 and its derivatives suggested a dual mode of action of arenicin-3 against Gram-negative bacteria. Arenicin-3 binds to and disrupts the integrity of both the outer and cytoplasmic membranes of Gram-negative bacteria through direct binding to phospholipids, independently of lipid A. Transposon directed insertion sequencing (TraDIS) data suggested that arenicin may interrupt phospholipid transportation pathways between the two membranes, leading to dis-regulation of membrane composition and compromised membrane integrity [16]. Moreover, it has been shown that an arenicin-3 derivative, NZ17074, killed the fungus *Candida albicans* by disrupting the cell membrane, inducing apoptosis and interrupting the cell cycle [17]. Huang et al. [16] showed that AA230 (also known as NZ17230) did not show haemolysis of human red blood cells even at a concentration of 300 µg/mL, which is markedly higher than the MIC/MBC.

Another aim of this study was to examine the antibacterial activity of EDTA. Our results are generally in agreement with the results obtained by Hamoud et al. [29], demonstrating that EDTA has only bacteriostatic activity against Gram-negative bacteria. EDTA has also been shown to exert a bacteriostatic effect against Gram-positive bacteria [29]. EDTA acts on the cell surface, resulting in the rapid release of approximately half of the lipopolysaccharide with a negligible loss of other cell components [30]. The disruption of the lipopolysaccharide structure in the outer membrane of Gram-negative bacteria occurs because EDTA chelates divalent cations. The release of lipopolysaccharides increases the membrane permeability to other agents, hence the potentiating action [21,30,31,32,33,34,35]. EDTA was found to affect the growth of *P. aeruginosa*, revealing a biphasic inhibitory pattern that suggests different mechanisms of action at different concentrations [21]. It has been postulated that at low concentrations a general inhibitory process occurs because of the removal of metal ions from the inhibitory medium [21]. Indeed, Richards and Cavill [36] showed that at low concentrations EDTA caused the formation of convoluted surfaces, but not lysis. Our time-kill results suggest that such a behavior is likely to occur against *P. aeruginosa* and *E. coli* strains and, at lower doses of EDTA, against *A. baumannii*. Nevertheless, at higher concentrations, EDTA can destabilize the outer membrane to such an extent that it may lead to cell lysis [21]. Both *A. baumannii* strains were more susceptible to EDTA than *E. coli* or *P. aeruginosa*. The important decrease in colony counts that was observed for both *A. baumannii* strains (even 3 log CFU/mL differences) may be attributed to the lysis of some bacterial cells at higher EDTA concentrations due to membrane destabilization.

EDTA has been shown to increase the permeability of Gram-negative bacteria to a variety of compounds, including some antibiotics [32]. It has been shown that EDTA enhances the effect of antibiotics such as ampicillin, penicillin G, tetracycline and chloramphenicol on a resistant strain of *P. aeruginosa* due to synergism of the antibiotic/EDTA combinations [37]. The resistance of *Pseudomonas aeruginosa* to these antibiotics can be reversed through the combined use of EDTA with these antibiotics [37]. Because in some cases resistance to antibiotics can be a consequence permeability barrier development, which prevents antibiotics from reaching their site of action, it has been suggested that the synergistic effect of EDTA/antibiotic combinations and the reversal of antibiotic resistance occurs due to changes in bacterial permeability produced by EDTA [32,37]. EDTA had also been shown to act in synergy with a 12-residue cationic antimicrobial peptide ARVA [3]. Our study showed that a significant dose reduction (2–4-fold) of AA230 can be achieved when used in combination with EDTA. Importantly, the synergy between EDTA and AA230 was also seen in multidrug resistant clinical isolates.

There are essentially two pathways that antibacterial molecules can take through the outer membrane: A lipid-mediated pathway for hydrophobic molecules and general diffusion porins for hydrophilic antibiotics [38]. Polymyxin B is an antibiotic composed of several closely related peptides with a molecular weight of 1301.56 g/mol. It resembles AA230 in that it is an amphipathic molecule for which the cytoplasmic membrane is the target. For binding to the cytoplasmic membrane to occur, the molecule (either polymyxin or AA230) must cross the outer membrane barrier. Since polymyxin B is too large to go through the narrow porin channel of enteric bacteria [39], it can be concluded that AA230, which is a larger molecule than polymyxin B, is not capable of going through the porin channel either. Polymyxin B is thought to gain access to the cytoplasmic membrane through binding to the outer membrane and causing alterations, disorganization and disruption [39,40,41]. It is very likely that a similar mechanism is involved in the case of AA230. Interestingly, it was shown that the activity of polymyxin B against *P. aeruginosa* was substantially increased in the presence of EDTA as a result of permeability barrier reduction [20]. The synergy between AA230 and EDTA observed in our study could be attributed to the fact that the EDTA disturbs the permeability of the outer membrane, thereby allowing a higher influx of AA230 into the bacterial cell, which leads to an increase in AA230 activity.

The results obtained in this work suggest several avenues of new research. Combinations of arenicin-3 derivatives with EDTA could be of real benefit for topical applications against Gram-negative bacteria, including strains resistant to conventional antibiotics. Investigation into interactions between arenicin-3 derivatives and other chelating agents including antibiotics such as ciprofloxacin or research into the development of new biocompatible chelating agents would also seem a reasonable suggestion of the path forward.

## 5. Conclusions

Antibacterial susceptibility testing indicated that AA230 exerted potent inhibitory effects against all of the tested bacterial strains regardless of their resistance patterns. AA230 exhibited a bactericidal mode of action, which suggests that AA230 has potential as a therapeutic agent for the treatment of bacterial infections, including those caused by antibiotic-resistant strains. Furthermore, EDTA increased the antibacterial activity of AA230. Hence, the addition of EDTA could enable the use of lower AA230 concentrations. The AA230/EDTA combinations could have potential applications in the treatment of infections caused by Gram-negative organisms, particularly *A. baumannii*, which was more susceptible to EDTA than *E. coli* and *P. aeruginosa*.

## Figures and Tables

**Table 1:** Minimum inhibitory concentrations (MICs) and minimum bactericidal concentrations (MBCs) of antimicrobial peptide AA230 and EDTA (ethylenediaminetetraacetic acid) against different strains of *P. aeruginosa*, *A. baumannii* and *E. coli*. MICs and MBCs are expressed in µg/mL.

Bacteria	MIC of AA230 (µg/mL)	MBC of AA230 (µg/mL)	MIC of EDTA (µg/mL)	MBC of EDTA (µg/mL)
*A. baumannii* AYE	2–4	4	1024	≥16,384
*A. baumannii* RCH	2–4	4–8	512	≥16,384
*E. coli* ATCC	2–4	2–4	≥16,384	≥16,384
ESBL *E. coli*	1–2	1–2	≥16,384	≥16,384
*P. aeruginosa* ATCC	4	8–16	8192	≥16,384
*P. aeruginosa* clinial	4–8	8	8192	≥16,384

**Table 2:** Minimum inhibitory concentrations (MICs) and fractional inhibitory concentration (FIC) indexes of antimicrobial peptide AA230 and EDTA in combination against different strains of *P. aeruginosa*, *A. baumannii* and *E. coli*. MICs are expressed in µg/mL. S: synergy; A: additivity.

MIC AA230 in Combination (µg/mL)	MIC EDTA in Combination (µg/mL)	FIC Index	Interpretation
*E. coli* ATCC			
0.5	64	0.129–0.254	S
1	64	0.254–0.504	S
ESBL *E. coli*			
0.25	128	0.133–0.258	S
0.5	64	0.254–0.504	S
*A. baumannii* ATCC			
0.5	256	0.375–0.500	S
1	64	0.313–0.563	S/A
*A. baumannii* RCH			
0.5	256	0.625–0.750	A
1	128	0.500–0.750	S/A
*P. aeruginosa* ATCC			
2	128	0.516	A
*P. aeruginosa* clinical			
2	256	0.281–0.531	S/A
4	128	0.516–1.016	A
